# Effects of polysaccharides from *Lyophyllum decastes* (Fr.) Singer on gut microbiota *via in vitro*-simulated digestion and fermentation

**DOI:** 10.3389/fmicb.2023.1083917

**Published:** 2023-02-13

**Authors:** Fangfang Zhang, Ying Xiao, Liang Pan, Ling Yu, Yanfang Liu, Deshun Li, Xiaojie Liu

**Affiliations:** ^1^School of Perfume and Aroma Technology, Shanghai Institute of Technology, Shanghai, China; ^2^School of Health and Social Care, Shanghai Urban Construction Vocational College, Shanghai, China; ^3^National Engineering Research Center of Edible Fungi, Key Laboratory of Edible Fungi Resources and Utilization (South), Ministry of Agriculture and Rural Affairs, Shanghai, China; ^4^Institute of Edible Fungi, Shanghai Academy of Agricultural Sciences, Shanghai, China

**Keywords:** *Lyophyllum decastes* (Fr.) Singer, polysaccharides, simulated digestion, fermentation, short-chain fatty acids

## Abstract

**Introduction:**

*Lyophyllum decastes* (Fr.) Singer polysaccharides (LDSPs) have been verified to possess strong biological properties. However, the effects of LDSPs on intestinal microbes and their metabolites have rarely been addressed.

**Methods:**

The *in vitro*-simulated saliva-gastrointestinal digestion and human fecal fermentation were used to evaluate the effects of LDSPs on non-digestibility and intestinal microflora regulation in the present study.

**Results:**

The results showed a slight increase in the content of the reducing end of the polysaccharide chain and no obvious change in the molecular weight during *in vitro* digestion. After 24 h *in vitro* fermentation, LDSPs were degraded and utilized by human gut microbiota, and LDSPs could be transformed into short-chain fatty acids leading to significant (*p* < 0.05) decrease in the pH of the fermentation solution. The digestion did not remarkably affect the overall structure of LDSPs and 16S rRNA analysis revealed distinct shifts in the gut microbial composition and community diversity of the LDSPs-treated cultures, compared with the control group. Notably, the LDSPs group directed a targeted promotion of the abundance of butyrogenic bacteria, including *Blautia*, *Roseburia*, and *Bacteroides*, and an increase in the n-butyrate level.

**Discussion:**

These findings suggest that LDSPs might be a potential prebiotic to provide a health benefit.

## 1. Introduction

*Lyophyllum decastes* (Fr.) Singer, commonly known as the fried chicken mushroom, has been recognized as a culinary delicacy in Asia with potential for the commercial culture and great economic importance (Arana-Gabriel et al., 2018). Apart from excellent flavor and texture, *Lyophyllum decastes* (Fr.) Singer is currently of great interest due to being a biologically rich source of various active substances (Pokhrel and Ohga, 2007). It is deserved to be mentioned that polysaccharides from *Lyophyllum decastes* (Fr.) Singer (LDSPs) exhibit a range of possible health benefits, including antioxidant, anticholinesterase, antibacterial, and antidiabetic (Miura et al., 2002; Pushpa and Purushothama, 2010; Tel et al., 2015; Deveci et al., 2021).

Increasing evidence demonstrates that the consumption of fungal-derived polysaccharides can result in specific changes in gastrointestinal microbiota. The composition and homeostasis of the gut microbial community have an intense and intimate linkage to human health and play a crucial role in a multitude of physiological functions involving metabolic, immunologic, and protective activities (Slavin, 2013). Polysaccharides derived from *Ganoderma lucidum* have been shown to alleviate rat Dextran sulfate (DSS)-induced colitis by stimulating the growth of beneficial bacteria, such as *Ruminococcus_1*, and reducing pathogens, such as *Escherichia-Shigella* (Xie et al., 2019). In addition, acting as a substrate for gut microorganisms, polysaccharides from fungi can facilitate gut microbiota to produce short-chain fatty acids (SCFAs) providing a health benefit in immune and metabolic disease (Sanna et al., 2019). For instance, *Poria cocos* polysaccharides significantly enhanced glucose and lipid metabolism and mitigated hepatic steatosis in ob/ob mice by elevating the levels of butyrate and butyrate-producing bacteria *Lachnospiraceae* and *Clostridium* (Sun et al., 2019). It was observed that the promising prophylactic and therapeutic properties of fungal polysaccharides might be attributed to their diversified abilities to restore gut microbial balance.

Meanwhile, different polysaccharides of fungal origin may possess distinct prebiotic properties, which could be critical to comprehend their modulation of gut microbiota composition (Jayachandran et al., 2018). An *in vitro* batch culture fermentation study examined the impact of edible mushrooms polysaccharides on aging gut microbiota characteristics, demonstrating that *Pleurotus* spp. and *C. Cylindracea* mushrooms induced a significant bifidogenic effect, while *P. eryngii* mushrooms simulated the growth of *Lactobacillus* spp. and butyrate-producing bacteria such as *F. prausnitzii* and *E. rectale/Roseburia* spp. group (Mitsou et al., 2020). Undeniably, fungal polysaccharides represent a vast and still untapped source assumed to be applied, but there is little known about the regulation of LDSPs on the intestinal microbiome and their metabolites.

We hypothesized that LDSPs should be neither digested nor absorbed; therefore, LDSPs could undergo fermentation in the colon and be utilized by the gut microbiota accompanied by changes in SCFAs. In this study, the simulated saliva-gastrointestinal digestion model was conducted to reveal the digestion behavior of LDSPs and the main changes in molecular weight (Mw) and the content of the reducing end of the polysaccharide chain (RC). *In vitro* anaerobic fermentation was performed, 16S rRNA sequencing was adopted to clarify the effect of LDSPs on gut microbial composition, and their fermentation characteristics were investigated by determining SCFAs production and pH value. This study was intended to provide evidence of the possible digestion and fermentation mechanism of LDSPs for application in new potential prebiotics.

## 2. Materials and methods

### 2.1. Materials and reagents

*Lyophyllum decastes* (Fr.) Singer was collected from an agricultural product processing base in Taixing, Jiangsu Province, China. The enzymes, used in this study, including α-amylase (100 U/mg), pepsin (3,000 U/g), and pancreatic enzyme (4,000 U/g), were purchased from Sigma-Aldrich (St. Louis, United States). SCFA standards including acetic, propionic, butyric, valeric, isobutyric, and isovaleric acids were purchased from Aladdin (Shanghai, China). All other chemicals and solvents used were analytical grade.

### 2.2. Preparation of the LDSPs

The *Lyophyllum decastes* (Fr.) Singer was prepared using the method described by Wu et al. (2017) with slight modifications. Hot water extraction was used to obtain polysaccharides from *Lyophyllum decastes* (Fr.) Singer. In brief, the dried *Lyophyllum decastes* (Fr.) Singer powders (50.0 g) were extracted three times with ultrapure water (1:15, w/v) at 100°C for 1 h. Then, polysaccharides extracted from *Lyophyllum decastes* (Fr.) Singer was further concentrated and ultra-filtered (molar mass cutoff, 5.0 kDa) to remove impurities and finally freeze-dried.

### 2.3. Determination of physiochemical characteristics of LDSPs

The protein content, total phenol content, and total carbohydrate content of LDSPs were determined by the Bradford method, Folin–Ciocalteu method, and phenol–sulfuric acid method (Dubois et al., 1951; Bradford, 1976). The monosaccharide composition of LDSPs was according to Wang et al. (2019) with some modifications. Here, 2 M aqueous trifluoroacetic acid (TFA) was used to hydrolyze LDSPs at 110°C for 4 h. Then, High-Performance Anion-Exchange Chromatography with Pulsed Amperometric Detection (HPAEC-PAD; Dionex ICS-5000+) equipped with the Dionex CarboPac PA20 analytical column (3 × 150 mm) was conducted to detect monosaccharides in hydrolyzed samples.

The Mw of LDSPs was determined using the modified method as previously reported (Chen G. et al., 2017) by high-performance size exclusion chromatography, equipped with a multi-angle laser light scattering and a refractive index detector (HPSEC-MALLS-RID Shimadzu, Kyoto, Japan). The separation of samples was applied on TSK-GEL G6000PWXL and G4000PWXL column (7.8 × 300 mm, TOSOH Crop., Tokyo, Japan). The sodium nitrate solution (0.15 mol/ml) was eluted and the flow rate was 0.5 ml/min, and the injection volume of the sample was 100 μl.

### 2.4. *In vitro*-simulated salivary-gastrointestinal digestion

The simulated *in vitro* digestion was performed according to the previously published procedures (Brodkorb et al., 2019) with minor modifications. First, 25 mg of LDSPs were dissolved in 5 ml of ultrapure water. Subsequently, 4 ml of simulated salivary fluid (SSF) was preheated at 37°C in a water bath and then added into a 5 ml of LDSPs solution (10 mg/ml), CaCl_2_ solution (0.025 ml, 0.3 M), and α-amylase solution (75 U/ml, 0.5 ml). Ultrapure water was added to supplement the solution to 10 ml to mix LDSPs solution with SSF to achieve a final ratio of 1:1 (wt/wt). In addition, the ultrapure water and inulin solution (10 mg/ml) added to the simulated digestion medium were, respectively, used as the blank control (CON) group and positive control (INU); then, each group was kept in a 37°C shaking bath for 5 min.

Thereafter, the pH was adjusted to 3.0 with HCl (6 mol/L). Afterward, 8 ml of simulated gastric fluid (SGF) was preheated at 37°C in a water bath, followed by the addition of the previous stage of 10 ml simulated digestive fluid, CaCl_2_ solution (0.01 ml, 0.3 M), pepsin solution (2000 U/ml, 1 ml), and ultrapure water to supplement the solution to 20 ml to achieve a final ratio of 1:1 (wt/wt). The simulated digestion samples were incubated at 37°C. During the digestion process, an equal volume of simulated gastric fluid was taken out at the time points of 0, 1, 2, and 4 h for further analysis.

Next, the pH was adjusted to 7.0 with NaOH (6 mol/L). Next, 12 ml of simulated intestinal fluid (SIF) was preheated in a 37°C water bath, followed by the addition of the previous stage-simulated digestive fluid, bile salt solution (2.5 ml, 10 mM), CaCl_2_ solution (0.04 ml, 0.3 M), a pancreatic enzyme solution (0.5 g, 100 U/ml), and ultrapure water to supplement it to 40 ml. During the digestion process, an equal volume of simulated gastric fluid was taken out at the time points of 0, 1, 2, 4, and 6 h for further analysis.

### 2.5. Collection and preparation of microbiota inoculums and *in vitro* fermentation

The *in vitro* fermentation was performed based on the previous method with minor modifications (Lam et al., 2018). First, fresh fecal samples were collected from six healthy volunteers (three women and three men, 20–30 years of age) who maintained a regular diet and had not received antibiotics or prebiotic treatment for 3 months. The fecal samples were immediately mixed with sterilized 0.1 M phosphate-buffered saline (pH 7.0) to produce a 10% (w/v) fecal suspension. Then, 2.0 g of peptone, 2.0 g of yeast extract, 0.5 g of cysteine-HCl, 0.1 g of NaCl, 2.0 g of NaHCO_3_, 0.04 g of K_2_HPO_4_, 0.04 g of KH_2_PO_4_, 0.01 g of MgSO_4_ 7H_2_O, 0.01 g of CaCl_2_ 6H_2_O, 0.02 g of hemin, 0.5 g of bile salt, 2.0 ml of Tween 80, 10 μl of vitamin K_1_, and 1.0 ml of resazurin 1% (w/v) were dissolved in 1 L ultrapure water to obtain a basic medium. Finally, LDSPs were selected as 1% (w/v) carbon sources, and the medium without carbon sources and 1% (w/v) inulin were used as a blank control (CON) and positive control (INU). The medium was adjusted to pH 7.0 using 1 mol^−1^ HCl and placed in an anaerobic chamber at 37°C overnight to pre-reduce the media. Here, 1.0 ml of 10% (w/v) fecal slurry was added to a 9.0 ml medium and placed in the 37°C anaerobic chamber and incubated for 0, 6, 12, and 24 h. Then, the samples were collected immediately and stored at −80°C for further analysis.

### 2.6. Determinations of LDSPs variations during *in vitro* digestion and fermentation

The reducing end of the polysaccharide chain (RC) contents of the digestion and fermentation products were analyzed by the dinitrosalicylic acid (DNS) method using glucose as the standard (Li et al., 2020). The Mw and residual carbohydrate of the digestion and fermentation products were determined according to the previous method.

### 2.7. The pH value and SCFAs analysis during *in vitro* fermentation

The pH of the fermentation system was measured with a standard pH meter (DELTA320, Mettler Toledo Co., Ltd. Shanghai, China). SCFAs were extracted by absolute ether following the method of Bai et al. (2021) and were analyzed by gas chromatography–mass spectrometry (GC–MS)-TQ8040 (Shimadzu, Kyoto, Japan) equipped with SH-Rtx®-WAX column (30 m × 0.25 mm i. d.; film thickness 0.25 μm). In brief, the fermented solution was centrifuged at 6000 *g* for 10 min. The 20 μl of 10% H_2_SO_4_ was added to acidify 500 μl of supernatant, and 500 μl of absolute ether was used to extract SCFAs. The mixtures were then centrifuged at 8000 *g* for 10 min at 4°C, and the phases were separated. The supernatant was taken and filtered using a 0.20 μm filter into a sample injection bottle. The temperature increased raised to 140°C at 7.5°C/min and held for 4 min, which then raised to 200°C at 60°C/min. Carrier gas helium was employed, the flow rate was 2.0 ml/min, the full scan mode in the m/z range was 20.0–300.0, and the injection volume was 1 μl. SCFA concentration was determined by the external standard method with corresponding standards.

### 2.8. Analysis of the gut microbiota

After fermentation of 24 h, high-throughput sequencing technology of bacterial 16S rRNA was implemented to investigate the impact of polysaccharides on the gut microbiota. Each sample of 24 h blank control fermentation group (CON group), 24 h inulin fermentation group (INU group), and 24 h *Lyophyllum decastes* (Fr.) Singer fermentation group (LDSPs group) was extracted using Qiagen QIAamp Fast DNA Stool Mini Kit, according to the manufacturer’s instructions. The DNA extraction from all samples was visualized on 1% agarose gel electrophoresis. PCR amplification was performed using TransStart FastPfu DNA Polymerase, and the amplicons were purified using the AxyPrep DNA gel extraction kit (Axygen Bioscience, Union City, United States) and quantified using QuantiFluor™-ST fluorometer (Promega, Madison, United States). The major PCR products from the V3–V4 region of the 16S rRNA gene amplified with primer pairs 338F (5′-ACTCCTACGGGAGGCAGCAG-3′) and 806R(5′-GGACTACHVGGGTWTCTAAT-3′) were sequenced on the Illumina Miseq platform by Shanghai Majorbio Bio-pharm Technology Co. Ltd. (Shanghai, China).

### 2.9. Statistical analysis

The microbiological data were analyzed on the online platform Majorbio Cloud Platform.^1^ First, the double-ended reads were quality-controlled and filtered according to the sequencing quality, and the optimization data after quality control (QC) splicing are obtained according to the overlapping relationship between the double-ended reads. Then, the sequence noise reduction method (DADA2/Deblur) is used to process the optimization data to obtain Amplicon Sequence Variant (ASV) representing sequence and abundance information. All data, including alpha diversity, beta diversity, and the examination of the bacterial taxonomic compositions, were obtained from ASV. By using one-way ANOVA, the microbiota substantial difference analysis was obtained.

The other experiments were carried out 5-fold. Data were expressed as mean ± standard deviation (SD). Statistical analysis was carried out using SPSS (Version 17.0, Chicago, United States). One-way analysis of variance (ANOVA) followed by Tukey’s test at a 5% confidence level was used to calculate the significant difference.

## 3. Results and discussion

### 3.1. Preliminary characterization of LDSPs

The basic physical and chemical properties of purified LDSPs are shown in Table 1. As shown in Table 1, the total carbohydrate content, total phenol content, and protein content of LDSPs were 90.51, 3.82, and 1.95%, respectively. Moreover, the monosaccharide composition of LDSPs was glucose (82.14%), fructose (14.20%), galactose (2.49%), mannose (0.82%), fucose (0.20%), arabinose (0.10%), and rhamnose (0.04%). These results indicated that the main monosaccharide of LDSPs was glucose. HPGPC chromatogram showed the two peaks with an average molecular weight of 4.59 × 10^7^ Da and 4.30 × 10^6^ Da (1.14:1).

**Table 1:** Basic physical and chemical properties of LDSPs.

	Parameters	Value
Essential component (%)	Carbohydrate	90.51 ± 0.12%
	Phenol	3.82 ± 0.09%
	Protein	1.95 ± 0.03%
Monosaccharide composition (%)	Glucose	82.14 ± 0.03%
	Arabinose	0.10 ± 0.01%
	Fucose	0.20 ± 0.02%
	Rhamnose	0.04 ± 0.00%
	Galactose	2.49 ± 0.01%
	Fructose	14.20 ± 0.02%
	Mannose	0.82 ± 0.00%

Data are expressed as mean ± SD (*n* = 5). LDSPs, *Lyophyllum decastes* polysaccharides.

### 3.2. Digestive stabilities of LDSPs during *in vitro*-simulated digestion

#### 3.2.1. Changes in the reducing end of the polysaccharide chain (RC) contents released from LDSPs

Salivary amylase and the severe pH in the simulated gastric fluid may play important roles in the digestion of non-starch polysaccharides (Zhang et al., 2021). Moreover, due to the presence of pancreatin and high concentration of bile acids, there may be changes in the chemical composition of polysaccharides after intestine digestion. After hydrolyzed by digestive fluid, the glycosidic of polysaccharides bonds is destroyed accompanied by the increase in reducing end. As shown in Table 2, the contents of RC in LDSPs did not change significantly after simulated saliva digestion, indicating that LDSPs were not affected by salivary amylase. During simulated gastric digestion and intestinal digestion, both LDSPs and INU were partially degraded, but RC content increased significantly (*p* < 0.05) in the INU group compared to the LDSPs group. Overall, the structures of LDSPs were not significantly influenced by simulated saliva-gastrointestinal fluid, so they could be neither digested nor absorbed and directed to the colon to be utilized by gut microbiota.

**Table 2:** Changes in reducing end of polysaccharide chain content during *in vitro* digestion.

	Time	Reducing end of polysaccharide chain content (mg/mL)		
		CON	INU	LDSPs
Saliva digestion	0 min	0.122 ± 0.004a	0.701 ± 0.005a	0.145 ± 0.004a
	5 min	0.132 ± 0.012a	0.709 ± 0.001a	0.146 ± 0.001a
Gastric digestion	0 h	0.148 ± 0.005a	0.389 ± 0.001c	0.159 ± 0.001b
	2 h	0.149 ± 0.002a	0.591 ± 0.006b	0.161 ± 0.005b
	4 h	0.155 ± 0.009a	0.808 ± 0.002a	0.165 ± 0.008a
Intestinal digestion	0 h	0.108 ± 0.002a	0.342 ± 0.001b	0.116 ± 0.005c
	2 h	0.110 ± 0.003a	0.346 ± 0.002b	0.118 ± 0.005bc
	4 h	0.112 ± 0.013a	0.350 ± 0.001b	0.120 ± 0.001ab
	6 h	0.109 ± 0.002a	0.370 ± 0.001a	0.121 ± 0.001a

Data are expressed as mean ± SD (*n* = 5), and a–d mean significantly different (*p* < 0.05) by a Tukey test in the same group with different time points. CON, the blank control (no additional carbon source supplement); INU, the positive control (INU supplement), and LDSPs, the experimental group (LDSPs supplement).

#### 3.2.2. Changes in molecular weight

The digestion properties of LDSPs are also related to their changes in molecular weights (Wu et al., 2018). The molecular weight distributions of two peaks during digestion are shown in Table 3 and Supplementary Figure 1A. After *in vitro*-simulated digestion, the molecular weights of LDSPs did not change significantly (*p* < 0.05), in agreement with the changes in RC, which indicated that LDSPs were indigestible during *in vitro* digestion.

**Table 3:** Changes in molecular weight of LDSPs during *in vitro* digestion and fermentation.

Samples	Peak 1	Peak 2
	Mw (Da)	Mw (Da)
LDSPs	4.59× 10^7^ ± 0.04a	4.30 × 10^6^ ± 0.15a
LDSPs-S	4.52× 10^7^ ± 0.12a	4.25 × 10^6^ ± 0.09a
LDSPs-G	4.46× 10^7^ ± 0.23a	4.21 × 10^6^ ± 0.19a
LDSPs-I	4.41× 10^7^ ± 0.09a	4.19 × 10^6^ ± 0.13a
LDSPs-6 h	4.08× 10^7^ ± 0.14b	3.96 × 10^6^ ± 0.18b
LDSPs-12 h	3.37× 10^6^ ± 0.16c	3.55× 10^5^ ± 0.09c
LDSPs-24 h	2.35× 10^6^ ± 0.10d	2.03× 10^5^ ± 0.24d

Data are expressed as mean ± SD (*n* = 5), and a–d mean significantly different (*p* < 0.05) by a Tukey test in the same group with different time points. LDSPs-S, LDSPs-G, and LDSPs–I: samples of LDSPs digested after different digestion fluids in vitro, including salivary, saliva-gastric, and saliva-gastrointestinal digestions, respectively; LDSPs-6, LDSPs-12, and LDSPs-24: LDSPs fermented by human fecal microbiota at different fermentation time points of 6, 12, and 24 h, respectively.

### 3.3. *In vitro* fermentation of indigestible LDSPs by human feces

#### 3.3.1. Changes in the RC and residual carbohydrate

**Figure 1:**
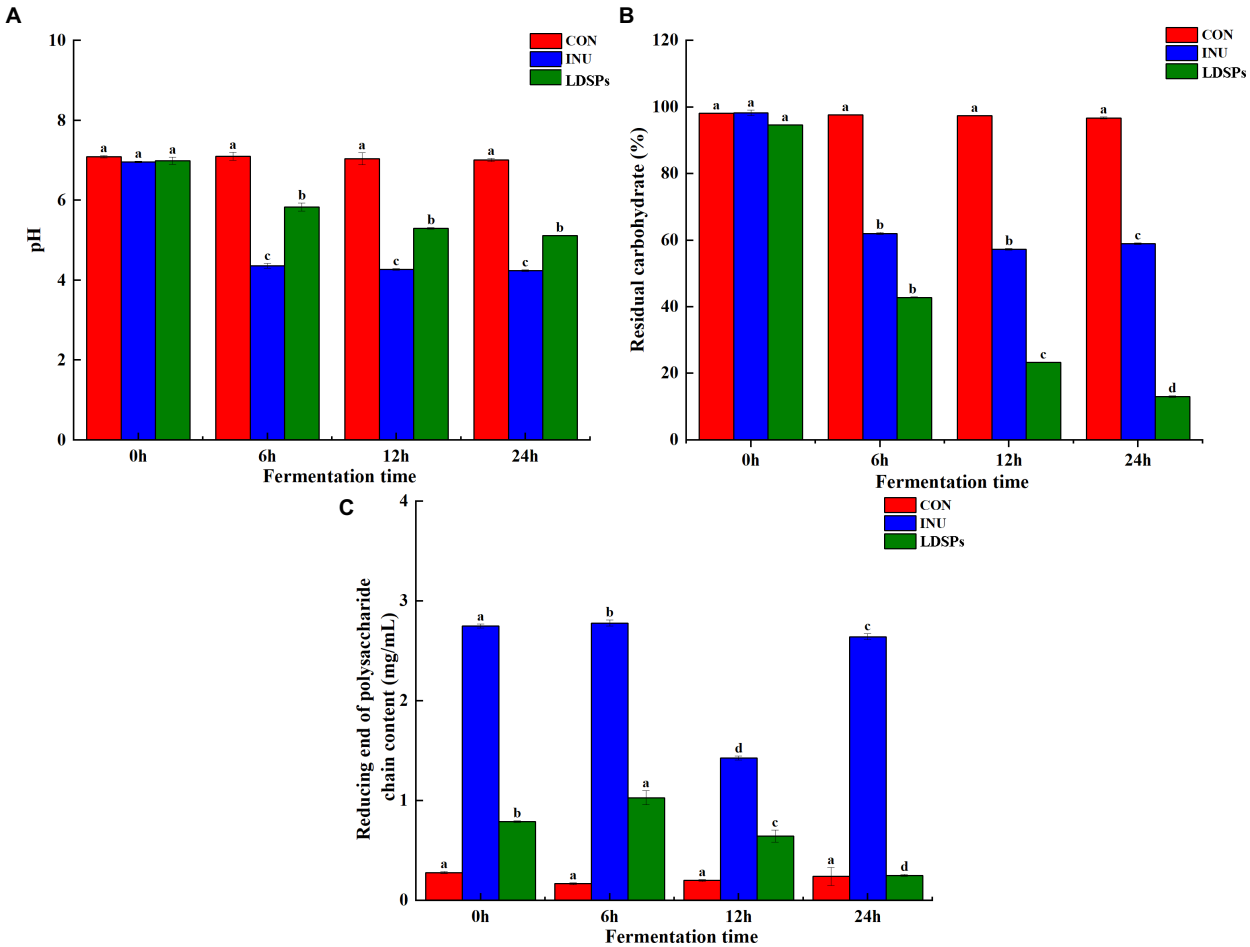
pH value **(A)**, residual carbohydrate **(B)**, and reducing end of polysaccharide chain content **(C)** during *in vitro* fermentation, respectively. Data are expressed as mean ± SD (*n* = 5), and a–d mean significantly different (*p* < 0.05) by a Tukey test in the same group with different time points.

*Lyophyllum decastes* (Fr.) Singer was subjected to an *in vitro* fermentation model inoculated with human fecal microbiota. Recently, the accumulating evidence shows that large polysaccharides are indigestible in saliva-gastrointestinal fluid for the lack of corresponding enzymes but can be degraded by the intestinal microbiota to increase the reducing end of the polysaccharide chain (Guo et al., 2021). As shown in Figure 1C, the RC and residual carbohydrate content were detected at 6, 12, and 24 h of fermentation. The LDSPs group showed a significant (*p* < 0.05) increase in the contents of RC during 0–12 h fermentation and a decrease after 12 h of fermentation. Combined with the sharp (*p* < 0.05) reduction in residual carbohydrates in Figure 1B, it was clear that the LDSPs were degraded into polysaccharide chains with reducing ends by the gut microbiota before the first 12 h. In addition, the degradation rate of LDSPs was higher than the utilization rate, and with the rapid proliferation of microbiota, the polysaccharide chains with reducing ends were easily utilized by gut microbes; therefore, RC was gradually decreased during the 12–24 h of fermentation. Indeed, the total residual carbohydrate remarkably (*p* < 0.05) decreased to 39.31 and 81.59% in INU and LDSPs groups, respectively, after the *in vitro* fermentation for 24 h, suggesting that they can be partially or fully hydrolyzed by intestinal microbiota, which is determined by the physicochemical properties of polysaccharides (Hu et al., 2018).

#### 3.3.2. Changes in molecular weight

The possible changes in molecular weight of LDSPs during *in vitro* fermentation were further investigated. Table 3 and Supplementary Figure 1B show the Mw changes in two peaks in LDSPs, and the Mw of LDSPs showed a slight decrease during 0–6 h and marginally decreased after 12 h of fermentation. These observations demonstrated that the microbiota went through a lag period in the initial 12 h and then degraded LDSPs quickly into polysaccharide chains with reducing ends for proliferation.

#### 3.3.3. Effects of LDSPs on gut microbiota

**Figure 2:**
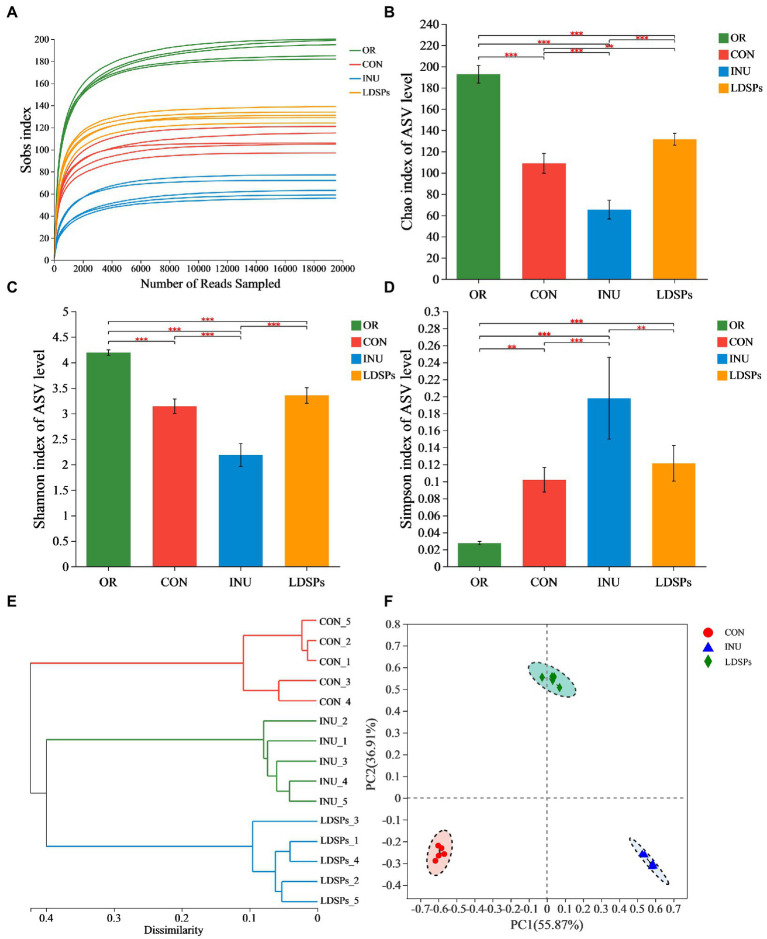
Alpha diversity analysis using the Student’s *t*-test for the Ace **(A)**, Chao **(B)**, Shannon **(C)**, and Simpson indices **(D)** and β-diversity analysis using UPGMA for hierarchical clustering **(E)** and ANOSIM for PCoA **(F)** on ASV level of gut microbiota in groups (*n* = 5) after 24 h of fermentation. *p < 0.05, **p < 0.01, and ***p < 0.001, respectively; OR, the initial sample group, CON, the blank control (no additional carbon source supplement), INU, the positive control (INU supplement), and LDSPs, the experimental group (LDSPs supplement); ANOSIM, analysis of similarities; PCoA, principal co-ordinate analysis.

In the present work, the high-throughput sequencing analysis was conducted on samples after 24 h fecal fermentation to reveal the effect of the indigestible LDSPs on the microbial structure. The average Good’s coverage was 99.28%, indicating the 16S rRNA sequences identified in this study likely represent the majority of bacterial sequences present in the samples. Diversity data analysis of 60 samples was completed and obtained ranging from 3,530,423 to 1,472,821,519 reads, with an average sequence length of 418 bp. The Sobs index, Shannon index, Simpson index, hierarchical clustering analysis, and principal co-ordinate analysis (PCoA) are shown in Figure 2. The rarefaction curves for the Sobs index exhibits the numbers of observed species per sample, and each sample reached plateaus, indicating that the majority of the sequencing was already sufficient (Figure 2A). Both the community diversity and richness in the LDSPs group significantly decreased after 24 h of fermentation compared with the initial sample group (OR) but higher than that in the 24 h blank group (CON) and the 24 h inulin supplement group (INU). Furthermore, the PCoA is an important analytical method for β-diversity. The PCoA score plot was used to reveal the microbiota shifted in both LDSPs and INU groups (Figure 2F). The total alternation of principal component 1 (PC1; 55.87%) and principal component 2 (PC2; 36.91%) was 92.78%. PCoA results show that both the LDSPs group and the INU group were far away from the CON group, and the outcome of hierarchical clustering analysis (Figure 2E) was in line with the PCoA results, indicating that LDSPs and INU could significantly affect the structure of the microbial community.

**Figure 3:**
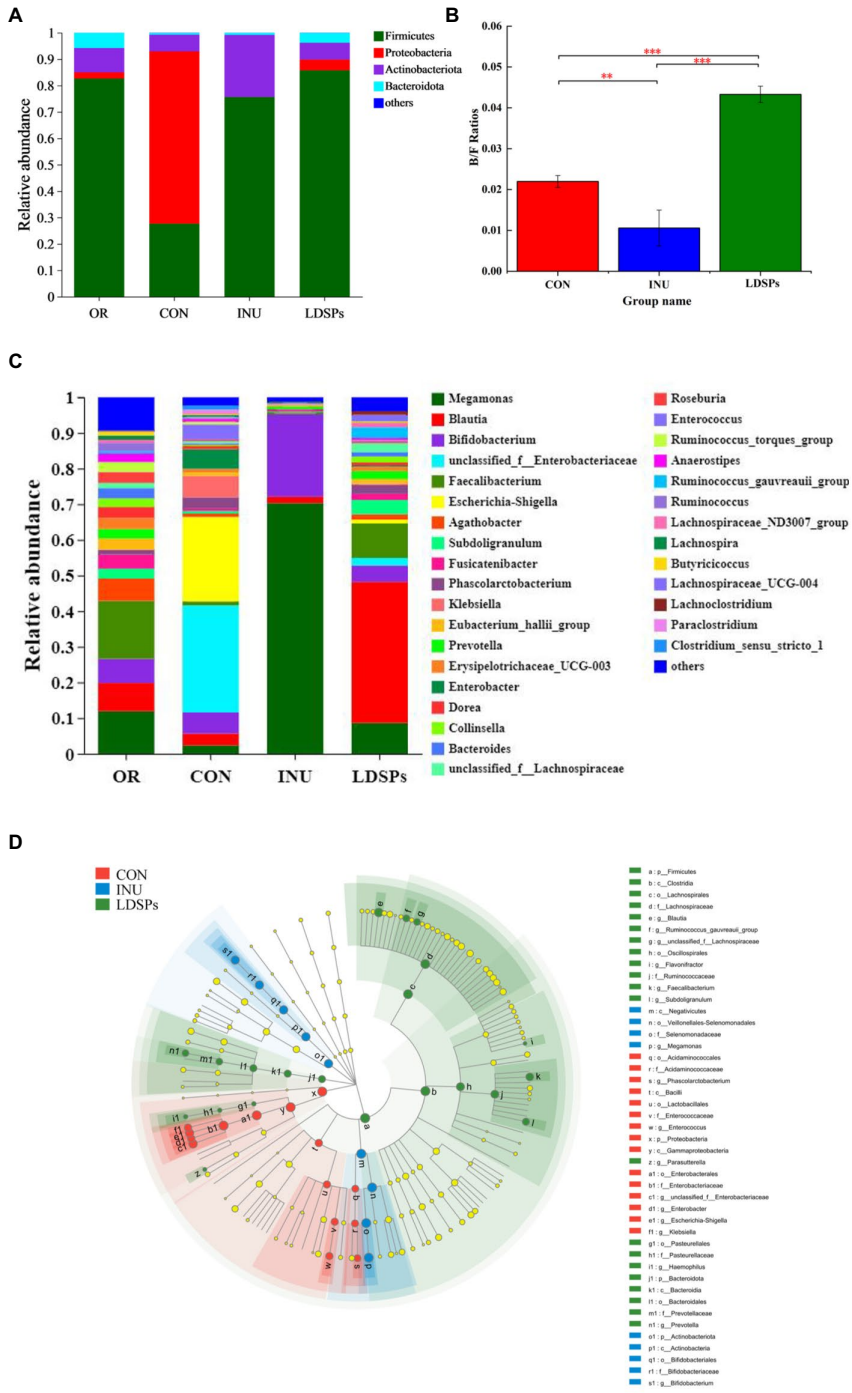
Relative abundance of gut microbiota community at the phylum **(A)** and genus **(C)** levels, *Bacteroidetes/Firmicutes* (B/F) ratios **(B)**, and the comparison of microbiota from phylum level to genus level among the CON, INU, and LDSPs groups based on linear discriminant analysis effect size (LEfSe; **D**) after 24 h of fermentation. *p < 0.05, **p < 0.01, and ***p < 0.001, respectively; OR, the initial sample group, CON, the blank control (no additional carbon source supplement), INU, the positive control (INU supplement), and LDSPs, the experimental group (LDSPs supplement).

At the phylum level, the dominant bacterial communities comprised *Firmicutes*, *Proteobacteria*, *Actinobacteria*, and *Bacteroidetes* (Figure 3A). Although the LDSPs group (85.75%) was noticeably richer in the relative abundance of *Firmicutes* compared with the CON group (27.66%), the *Bacteroidetes* to *Firmicutes* (B/F) ratio in the LDSPs group was significantly (*p* < 0.05) upregulated with 97% compared with the CON group (Figure 3B). *Bacteroidetes* is one of the major gut bacteria that could degrade polysaccharides, and the increased B/F ratio could alleviate obesity, which was considered one of the essential biological indicators (Ai et al., 2017). In the control group, an unsuitable ratio between protein and carbohydrate could result in increases in the number of potential pathogens due to disruption of the homeostasis of the gut micro-ecosystem with a higher abundance of *Proteobacteria* (Zhao et al., 2018). In addition, the LDSPs (4.10%) and INU (0.02%) also remarkably decrease the relative abundance of *Proteobacteria* compared with CON (65.28%), which might be attributed to the fact that LDSPs and INU could inhibit pathogens belonging to *Proteobacteria* such as *Escherichia-Shigella*. *Actinobacteria*, represented by the major probiotic bacteria *Bifidobacterium*, significantly increased in the INU group, compared with the CON group.

As shown in Figure 3C, three groups displayed different gut microbiota distributions at the genus level. The CON group was mainly composed of *Escherichia-Shigella* (23.72%), *unclassified_f_Enterobacteriacece* (30.02%), *Enterobacter* (5.51%), *Klebsiella* (6.02%), *Bifidobacterium* (5.94%), and *Phascolarctobacterium* (3.17%). However, *Megamonas* (70.30%) and *Bifidobacterium* (23.22%) became the dominant microbiota for the INU group after 24 h of fermentation, indicating that *Megamonas* might be the principal gut microbiota to degrade and utilize INU, similar to the result reported in a previous study (Chen B. D. et al., 2017). The LDSPs group also possessed higher levels of *Blautia* (39.59%), *Faecalibacterium* (9.69%), *Dorea* (3.62%), and *Subdoligranulum* (3.99%) than that of the CON group. The increasing works have suggested that *Blautia* is a kind of gut microbiota to promote the production of butyric acid (Cantu-Jungles et al., 2018).

The linear discriminant analysis effect size (LEfSe) in Figure 3D indicates the abundance of significantly different bacteria from the phylum level to the genus level in the CON, INU, and LDSPs groups. *Prevotella* has been reported to play an important role in glucose homeostasis and host metabolization (Si et al., 2017), and it was observed at a higher level in the LDSPs group than that in the CON and INU groups in this study. In addition, the increased *Prevotella* might be associated with the consumption of dietary fiber or carbohydrates (Kovatcheva-Datchary et al., 2015). Furthermore, *clostridium* and *Lachnospiraceae*, in the Firmicutes phylum, presented large increases in the LDSPs ferments and are known to be responsible for most of the butyrate produced in the human gut (Louis and Flint, 2009). The relative abundance of *Bifidobacterium* significantly increased in the INU group, which has been proved recently that could control serum cholesterol levels, prevent intestinal diseases, and modulate the immune system (Di Gioia et al., 2014). However, the lower abundance of *Bifidobacterium* in the LDSPs group, which might be a poor utilization of LDSPs by bifidobacterial, is consistent with the report that *Bifidobacterium* was not found in *in vitro* fermentation of polysaccharides from Fuzhuan brick tea (Chen G. et al., 2017). The probiotics can be broadly defined, and probiotics are live bacteria and yeasts, which are beneficial to human health when administrated in a viable form and in adequate amounts (Valdes et al., 2018). These results suggested that the commensal bacteria in the intestinal tract could break down indigestible LDSPs and that LDSPs could regulate gut microbiota dysbiosis by supporting the growth of beneficial bacteria and suppressing the proliferation of harmful bacteria, while the effects of LDSPs and INU on retarding dysbiosis were quite different.

#### 3.3.4. Effects of LDSPs on PH and SCFAs

Fermentation of polysaccharides by microbes in the colon results in the production of SCFAs, and SCFAs are involved in the reduction of gut pH levels (Thandapilly et al., 2018). As shown in Figure 1A, the pH values of the INU group and the LDSPs group were significantly (*p* < 0.05) lower than the 0 h after 24 h of fermentation. Furthermore, the pH values decreased with significant differences between the INU and LDSPs groups (*p* < 0.05), which might be related to their diverse chemical structure. A previous study showed that lower pH of the intestinal tract could promote the growth of probiotics and inhibit the reproduction of pathogens (Shoukat and Sorrentino, 2021). The concentrations of acetic acid, propionic acid, n-butyric acid, i-butyric acid, n-valeric acid, and i-valeric acid produced during *in vitro* fecal fermentation are shown in Table 4. Both INU and LDSPs groups noticeably promote the production of SCFAs, especially for the significant increase (*p* < 0.05) in propionic acid and n-butyric acid in the LDSPs group. The increased level of acetic acid was observed in the INU group.

**Table 4:** Changes in SCFAs contents produced during fermentation.

SCFAs(mmol/L)	Time	CON	INU	LDSPs
Acetic acid	0	2.76 ± 0.10d, A	2.76 ± 0.10d, A	2.76 ± 0.10d, A
	6	5.02 ± 0.12a, C	7.18 ± 0.17c, B	20.72 ± 0.05b, A
	12	4.57 ± 0.05c, C	11.75 ± 0.11b, B	20.09 ± 0.13c, A
	24	4.90 ± 0.23b, C	28.64 ± 0.17a, B	18.70 ± 0.01a, A
Propionic acid	0	0.86 ± 0.03d, A	0.86 ± 0.03d, A	0.86 ± 0.03d, A
	6	1.75 ± 0.10c, C	10.16 ± 0.29c, B	14.60 ± 0.09c, A
	12	3.06 ± 0.03b, C	11.71 ± 0.40b, B	17.78 ± 0.14b, A
	24	4.43 ± 0.02a, C	15.78 ± 0.20a, B	26.31 ± 0.16a, A
n-Butyric acid	0	0.76 ± 0.02c, A	0.76 ± 0.02d, A	0.76 ± 0.02d, A
	6	0.47 ± 0.05d, C	1.17 ± 0.13b, B	15.32 ± 0.18c, A
	12	1.80 ± 0.15a, C	1.57 ± 0.04a, B	34.44 ± 0.04b, A
	24	0.89 ± 0.07b, C	1.12 ± 0.01c, B	45.10 ± 0.29a, A
i-Butyric acid	0	ND	ND	ND
	6	0.06 ± 0.03b, B	ND	0.12 ± 0.05b, A
	12	0.20 ± 0.11a, A	0.03 ± 0.01, B	0.32 ± 0.01a, A
	24	0.19 ± 0.06a, B	0.13 ± 0.03, B	0.40 ± 0.09a, A
n-Valeric acid	0	ND	ND	ND
	6	0.21 ± 0.37c, A	ND	ND
	12	0.76 ± 0.66b, C	0.60 ± 0.21b, B	2.97 ± 0.19a, A
	24	1.83 ± 0.15a, B	1.18 ± 0.23a, A	1.91 ± 0.10b, A
i-Valeric acid	0	ND	ND	ND
	6	ND	ND	ND
	12	ND	1.00 ± 0.05a, B	1.42 ± 0.31a, A
	24	ND	ND	ND
Total acid	0	4.39 ± 0.12d, A	4.39 ± 0.12d, A	4.39 ± 0.12d, A
	6	7.46 ± 0.13c, C	18.51 ± 0.08c, B	50.64 ± 0.05c, A
	12	10.19 ± 0.16b, C	26.63 ± 0.19b, B	76.69 ± 0.09b, A
	24	12.05 ± 0.03a, C	46.72 ± 0.21a, B	92.02 ± 0.13a, A

Data are expressed as mean ± SD (*n* = 5), a–d (lowercase letter) mean significant differences among different times (*p* < 0.05) in the same group, and A–C (capital letter) represent significant differences among different groups (*p* < 0.05) at the same time. CON, the blank control (no additional carbon source supplement); INU, the positive control (INU supplement), and LDSPs, the experimental group (LDSPs supplement); ND, not detected.

Short-chain fatty acids have various positive effects on human health as prebiotic metabolites, and it has been confirmed that acetic acid is absorbed in the brain, heart, and peripheral tissues as an energy source (Kimura, 2014). Propionic acid produced in the intestinal tract improves tissue insulin sensitivity and suppresses cholesterol synthesis in the liver (Ding et al., 2019). In addition, butyric acid is the key to maintaining intestinal barrier integrity by providing energy for colonic epithelial cells and can also affect the host gene regulation, cell differentiation, and cell apoptosis (Ziegler et al., 2016). Moreover, the very high-level concentration of n-butyric acid exhibited in the LDSPs group might be primarily attributed to the relatively high abundance of *Firmicutes* according to previous reports (Fu et al., 2019).

**Figure 4:**
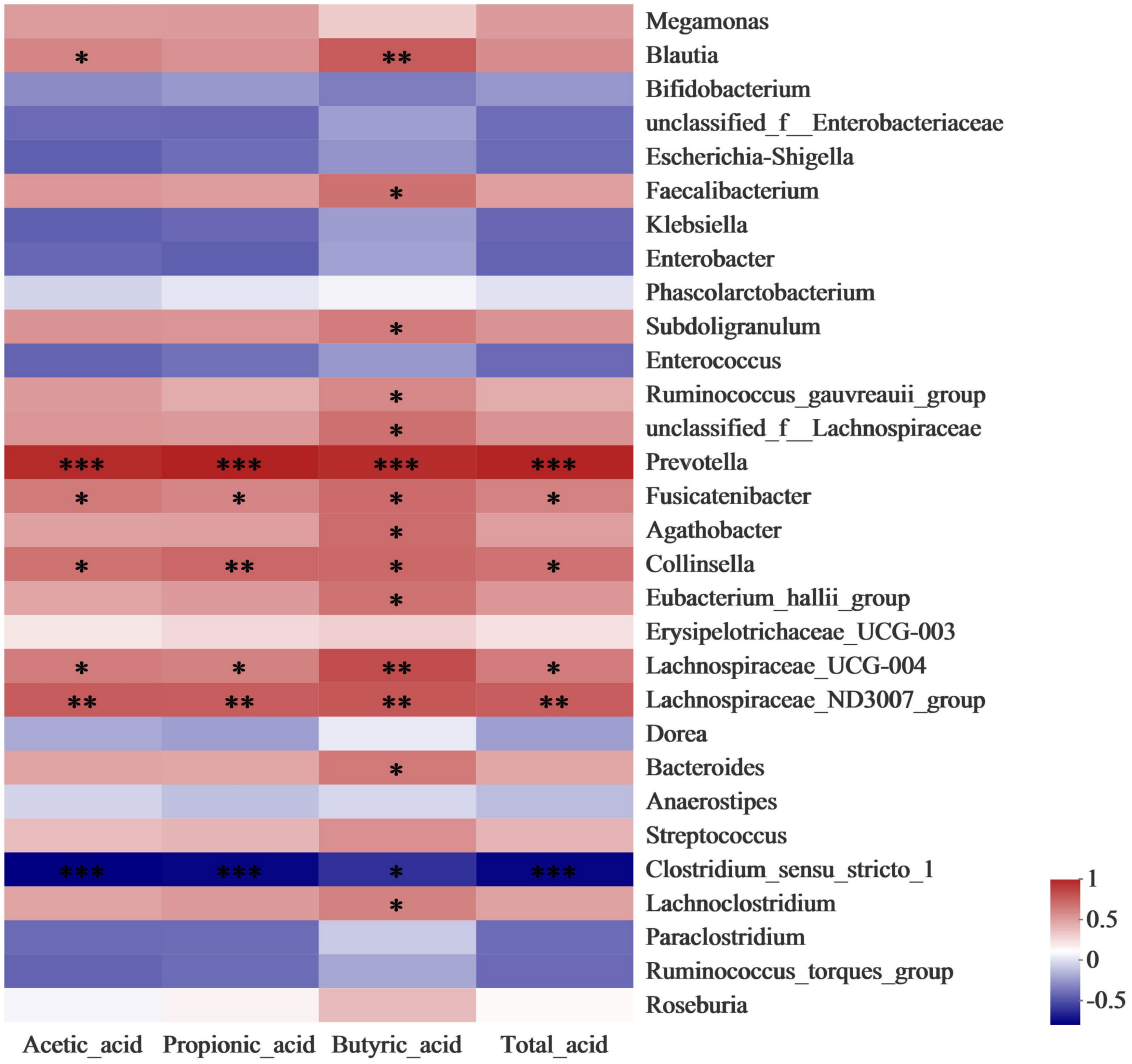
Heatmap analysis for correlation between gut microbiota community and SCFAs. *p < 0.05, **p < 0.01, and ***p < 0.001, respectively; CON, the blank control (no additional carbon source supplement), INU, the positive control (INU supplement), and LDSPs, the experimental group (LDSPs supplement); SCFAs, short-chain fatty acids.

Correlation analysis was conducted to further identify the relationship between the gut microbiota community and SCFAs. As shown in Figure 4, the level of butyrate has a significantly (*p* < 0.05) positive correlation to the abundance of *Blautia*, *Faecalibacterium, and Bacteroides*, which belong to members of butyrogenic *Clostridium cluster XIVa* (Cantu-Jungles et al., 2018). *Faecalibacterium* produces butyrate which is required for colonic epithelium repair and Treg cell production and plays a crucial role in human health (Faintuch and Faintuch, 2019). Moreover, the concentration of propionic acid was enhanced by the increase in the abundance of *Collinsella*. A previous study reported that the *Collinsella* genus correlated closely with the production of pro-inflammatory cytokine IL-17A and could ameliorate the permeability of the gut (Chen B. D. et al., 2017). The increases in the relative abundance of *Lachnospiraceae* and *Prevotella* in LDSPs was along with the promotion of total acid. *Lachnospiraceae* substantially involves the production of SCFAs that are positively correlated to the integrity of the epithelial barrier and immune activation, and its reduction in abundance is found in patients with Parkinson’s disease (Keshavarzian et al., 2020). The results provided strong evidence that LDSPs could exert health benefits by simulating the targeted abundance of beneficial bacteria to produce SCFAs.

## 4. Conclusion

In conclusion, we found that LDSPs were indigestible under simulated saliva-gastrointestinal digestion conditions and degraded and utilized by human gut microbiota after 24 h *in vitro* fermentation, resulting in a prominent increase in the concentration of SCFAs and a decrease in pH. Remarkably, the production of propionic acid and n-butyric acid after LDSPs fermentation causally correlated with the enrichments of *Prevotella*, *Blautia*, and *Lachnospiraceae*. Therefore, LDSPs may be a potential prebiotic for health benefits.

## Data availability statement

The data presented in the study are deposited in the NCBI repository (https://www.ncbi.nlm.nih.gov/bioproject/), accession number PRJNA930120.

## Author contributions

FZ: experimental studies, data analysis, and writing. YX: experiment design, conceptualization, project administration, and revision. LP: data curation and statistical analysis. LY: experiment design and resources. YL: funding acquisition and conceptualization. DL: experimental studies. XL: statistical analysis. All authors contributed to the article and approved the submitted version.

## Funding

This study was supported by the Shanghai Agriculture Applied Technology Development Program, China (grant no. X2021-02-08-00-12-F00797), and the Earmarked Fund for China Agriculture Research System, China (Grant No. CARS-20).

## Conflict of interest

The authors declare that they have no known competing financial interests or personal relationships that could have appeared to influence the work reported in this study.

## Publisher’s note

All claims expressed in this article are solely those of the authors and do not necessarily represent those of their affiliated organizations, or those of the publisher, the editors and the reviewers. Any product that may be evaluated in this article, or claim that may be made by its manufacturer, is not guaranteed or endorsed by the publisher.

## Footnotes

^1^ www.majorbio.com
