# Supplementary material for: Effects of polysaccharides from Lyophyllum decastes (Fr.) Singer on gut microbiota via in vitro-simulated digestion and fermentation
Source: Front Microbiol. 2023 Feb 13;14:1083917. doi: 10.3389/fmicb.2023.1083917 (PMC9969080; doi:10.3389/fmicb.2023.1083917)

**Supplemental Figure Captions**

**Supplemental Figure 1** Changes in high-performance size exclusion chromatograms of **LDSPs** in vitro digestion (A) and fermentation (B). **LDSPs**, *Lyophyllum decastes* polysaccharides. **LDSPs-S**, **LDSPs -G**, and **LDSPs -I**, samples of **LDSPs** digested after different in vitro digestion fluids, including salivary, saliva-gastric, and saliva-gastrointestinal digestions, respectively; **LDSPs-6**, **LDSPs-12**, and **LDSPs-24**, LDSPs fermented by human fecal microbiota at different fermentation time points of 6, 12, and 24 h, respectively.

**Supplemental Figure 1**
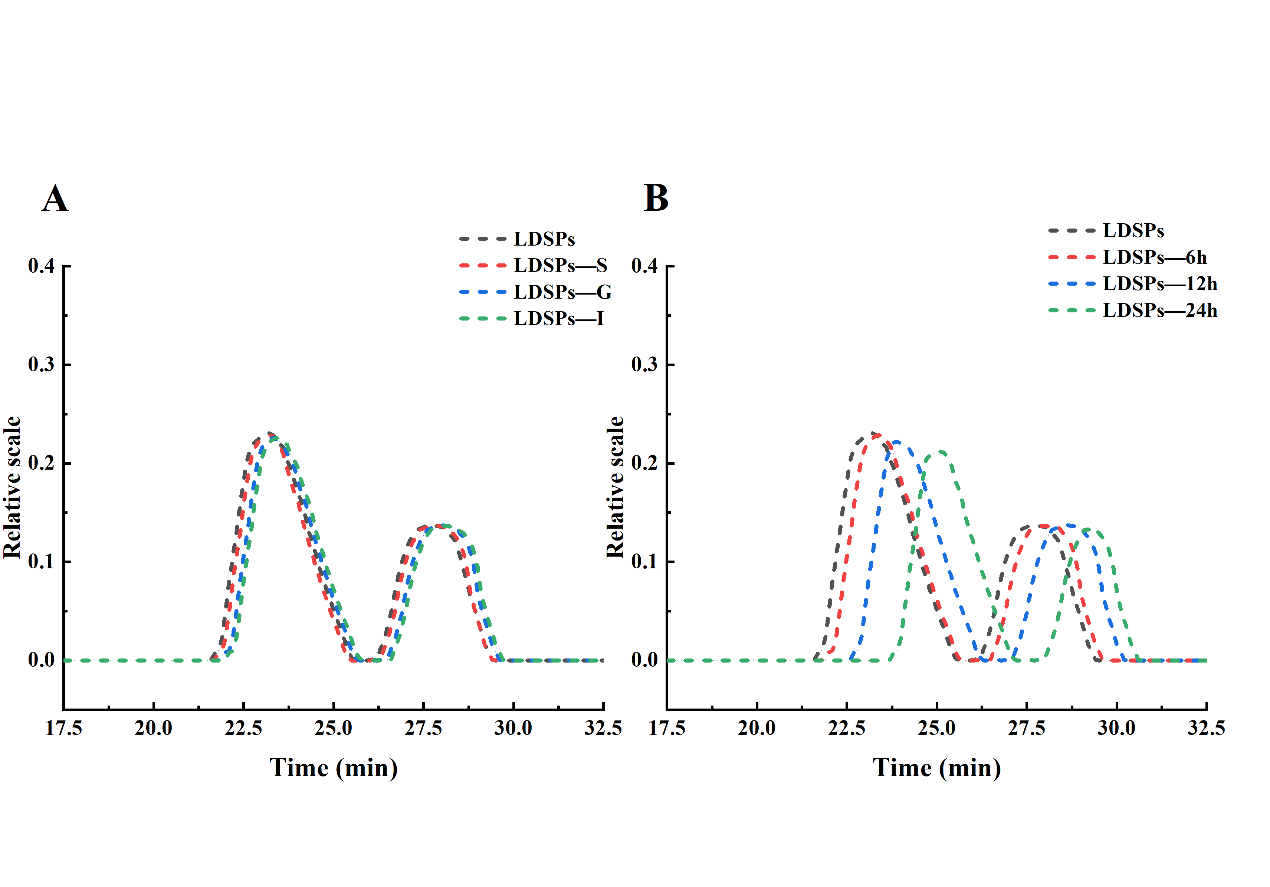

Supplement: Supplementary file 1 [file Data_Sheet_1.docx]
